# Posterior reversible encephalopathy syndrome (PRES) in a patient with moyamoya disease: A case report

**DOI:** 10.1097/MD.0000000000026837

**Published:** 2021-08-06

**Authors:** Chun-Hsin Teng, I-Hsiao Yang, Meng-Ni Wu, Ping-Song Chou

**Affiliations:** a Department of Neurology, Kaohsiung Medical University Hospital, Kaohsiung Medical University, Kaohsiung, Taiwan; b Department of Medical Imaging, Kaohsiung Medical University Hospital, Kaohsiung Medical University, Kaohsiung, Taiwan; c Department of and Master's Program in Neurology, Faculty of Medicine, College of Medicine, Kaohsiung Medical University, Kaohsiung, Taiwan; d Neuroscience Research Center, Kaohsiung Medical University, Kaohsiung, Taiwan.

**Keywords:** case report, hypertension, moyamoya disease, posterior reversible encephalopathy syndrome

## Abstract

**Introduction:**

Moyamoya disease (MMD) and posterior reversible encephalopathy syndrome (PRES) share similar pathophysiological characteristics of endothelial dysfunction and impaired cerebral autoregulation. However, there have never been any published studies to demonstrate the relationship between these 2 rare diseases.

**Patient concerns:**

A 26-year-old Asian man presented with a throbbing headache, blurred vision, and extremely high blood pressure. We initially suspected acute cerebral infarction based on the cerebral computed tomography, underlying MMD, and prior ischemic stroke. However, the neurological symptoms deteriorated progressively.

**Diagnosis:**

Cerebral magnetic resonance imaging indicated the presence of vasogenic edema rather than cerebral infarction.

**Interventions and outcomes:**

An appropriate blood pressure management prevents the patient from disastrous outcomes successfully. Cerebral magnetic resonance imaging at 2 months post treatment disclosed the complete resolution of cerebral edema. The patient's recovery from clinical symptoms and the neuroimaging changes supported the PRES diagnosis.

**Conclusion:**

This report suggests that patients with MMD may be susceptible to PRES. It highlights the importance of considering PRES as a differential diagnosis while providing care to MMD patients with concurrent acute neurological symptoms and a prompt intervention contributes to a favorable clinical prognosis.

## 1 Introduction

Moyamoya disease (MMD) is an idiopathic, chronic cerebrovascular disease characterized by progressively steno-occlusive changes at the distal internal carotid artery, with occasional middle cerebral artery and anterior cerebral artery involvements.^[1]^ Numerous small proliferative vessels developing at the base of the brain are collateral for the abovementioned stenotic vessels. They facilitate the unique “puff of smoke” look on digital subtraction angiography, officially named MMD by Suzuki and Takaku in 1969.^[2]^ MMD is a rare disease and incidence rates are higher in East Asian populations than European and North American populations.^[3]^ The clinical presentations of MMD vary with age and have a bimodal distribution. Ischemic strokes and transient ischemic attacks are common in children, whereas hemorrhagic strokes are predominant in adult patients.^[4]^

Posterior reversible encephalopathy syndrome (PRES) is a heterogeneous clinical and neuroradiological syndrome characterized by a parieto-occipital predominant pattern of reversible vasogenic edema. Symptoms include altered consciousness, headache, visual disturbances, and seizures.^[5]^ Clinical and radiological symptoms can be reversed with an appropriate diagnosis and management. Effective and early treatment is required for a favorable prognosis.

PRES has been reported to be associated with hypertension and endothelial dysfunction.^[6]^ Moreover, endothelial dysfunction and impaired cerebral autoregulation are reportedly pathophysiological characteristics of MMD.^[7,8]^ However, to the best of our knowledge, there is no study reporting the relationship between MMD and PRES. Here, we report on the serial clinical investigations and neuroimaging changes of a unique case presenting with MMD and PRES.

## 2 Case presentation

**Figure 1:**
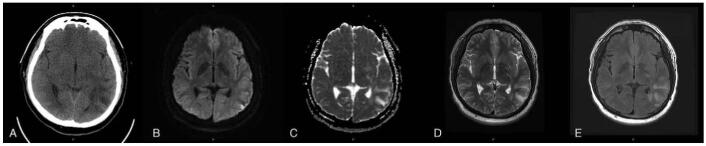
Cerebral computed tomography (A) disclosed a hypodense lesion in the left parieto-temporal lobe. Initially, it was difficult to differentiate between acute ischemic stroke and vasogenic edema. Cerebral MRI disclosed a slight hyperintense signal on diffusion-weighted imaging (B) in the left parieto-temporal lobe that was not accompanied by a decreased signal on apparent diffusion coefficient (C). Also, we identified hyperintensity of T2-weighted imaging (D) and fluid-attenuated inversion recovery (E) in the same area, which suggested the presence of vasogenic cerebral edema rather than cerebral infarction. MRI = magnetic resonance imaging.

A 26-year-old male, with a history of hypertension, prior ischemic stroke, and MMD, Suzuki stage 4, was prescribed acetazolamide, clonidine, amlodipine, and valsartan for hypertension control along with aspirin for secondary prevention of stroke. He presented to the emergency room with a worsening throbbing headache and blurred vision in January 2021. On arrival, he was afebrile, with a normal heart rate (89 beats per minute) but an extremely high blood pressure (202/140 mm Hg). The white blood cell count and C-reactive protein were within normal limits, and his renal and hepatic functions were normal. Both autoimmune and coagulopathy profiles were unremarkable, and there was no exposure to cytotoxic substances and/or immunosuppressants. The neurological examination revealed no focal neurological deficits, except blurred vision. However, cerebral computed tomography identified a hypodense lesion in the left parieto-temporal lobe, and thus acute cerebral infarction was initially impressed (Fig. 1A). The patient received the standard treatment for acute cerebral infarction, including antiplatelet therapy and maintaining blood pressure below 220/120 mm Hg. However, in the following days, the patient's systolic blood pressure was approximately 200 to 230 mm Hg, but the patient experienced altered consciousness, impaired memory, and irrelevant speech. Cerebral magnetic resonance imaging (MRI) reported hyperintensity of T2-weighted imaging and fluid-attenuated inversion recovery in the left parieto-temporal lobe. Diffusion-weighted imaging revealed a slight hyperintense signal. However, we did not identify a decreased signal on apparent diffusion coefficient, thereby suggesting the manifestation of vasogenic cerebral edema rather than a cerebral infarction (Fig. 1B–E). For suspected hypertension-related cerebral edema, the patient underwent a continuous intravenous infusion of nicardipine. This infusion and his previous oral anti-hypertension medications kept his systolic blood pressure between 140 and 160 mm Hg.

**Figure 2:**
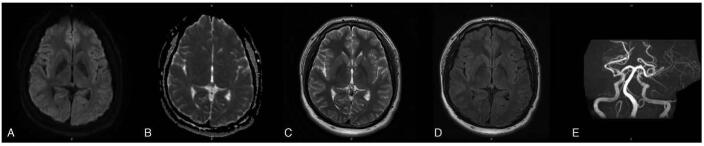
Follow-up cerebral MRI at 2 months post treatment revealed the complete resolution of cerebral edema in the left parieto-temporal lobe (A: diffusion-weighted imaging, B: apparent diffusion coefficient, C: T2-weighted imaging, D: fluid-attenuated inversion recovery). There was no change of moyamoya disease with stenotic bilateral distal internal carotid arteries, compared with previous neuroimages (E). The complete recovery of neuroimaging changes supported the diagnosis of PRES. MRI = magnetic resonance imaging, PRES = posterior reversible encephalopathy syndrome.

After we managed to control his hypertension for 1 week, the patient recovered consciousness, and intravenous nicardipine was replaced by oral medications. His clinical symptoms improved without neurological sequelae with a total hospitalization for 2 weeks. A follow-up cerebral MRI at 2 months posttreatment disclosed the complete resolution of cerebral edema without any newly developed intracranial lesion (Fig. 2). The complete recovery of clinical symptoms and neuroimaging changes supported the diagnosis of PRES. The patient remained asymptomatic for 2 months after discharge from the hospital and received left extracranial-intracranial bypass with encephaloduroarteriosynangiosis in March 2021.

## 3 Discussion

In this case study, we demonstrated a rare case of PRES predisposed by MMD, with a favorable outcome after opportune diagnosis and treatment. PRES could be triggered by acute hypertension and underlying endotheliopathy, which is associated with sepsis, pre-eclampsia, eclampsia, autoimmune disorders, and exposure to cytotoxic medications.^[9]^ According to our critical literature review, MMD has not been reported as a risk factor for PRES.

The histopathological findings of MMD revealed intimal thickening, the undulation of the elastic lamina, and attenuation of media.^[10]^ MMD vasculopathy could contribute to endothelial dysfunction, disruption of the blood–brain barrier, and impaired cerebral autoregulation.^[8]^ Cerebral autoregulation is the intrinsic ability of the brain to maintain constant cerebral perfusion despite alterations in arterial blood pressure.^[11]^ Therefore, vasculopathy facilitates a lower limit of blood pressure in patients with MMD to overwhelm the cerebral autoregulation threshold. This results in cerebral hyperperfusion and vasogenic edema, 2 main pathophysiologies of PRES.^[12]^

PRES is generally considered clinically and neuroradiologically reversible with a favorable prognosis. However, the prognosis depends on the etiologies, the time available to control the causative factor, the presence of intracerebral hemorrhage, and restrictive diffusion on MRI.^[13]^ However, patients with MMD have increased stroke risks, which may complicate the diagnosis and treatment for PRES. It is difficult to differentiate acute cerebral infarction and vasogenic edema from cerebral computed tomography. By contrast, MRI can differentiate PRES from early cerebral ischemia. As opposed to the hyperintense signal on diffusion-weighted imaging with a corresponding decreased signal on apparent diffusion coefficient in acute cerebral infarction, PRES usually presents with prominent vasogenic edema discernable in T2-weighted images and fluid-attenuated inversion recovery, with an isointense or hyperintense signal on diffusion-weighted imaging that is not accompanied by a corresponding decreased signal on apparent diffusion coefficient.^[6]^

In patients with MMD and acute hypertension, blood pressure must be lowered gradually to reach the mean arterial pressure to 105 to 125 mm Hg.^[13]^ However, in patients with acute cerebral infarction, blood pressure should be maintained below 220/120 mm Hg. In our case, we suspected acute cerebral infarction initially because of the underlying MMD, the history of cerebral infarction, and the cerebral computed tomography. Therefore, we were not to control blood pressure intensively. However, neurological symptoms deteriorated progressively during the hospitalization period. Cerebral MRI suggested the presence of vasogenic edema rather than cerebral infarction. Consequently, we gradually reduced blood pressure and the patient managed to recover completely without sequelae. Due to the different goals of blood pressure control between acute cerebral infarction and PRES, an appropriate diagnostic tool to differentiate between these 2 conditions and prompt management of blood pressure plays a crucial role in preventing the development of neurological sequelae.

In conclusion, this report suggests that patients with MMD could be susceptible to PRES due to the altered capacity for cerebral autoregulation. Also, we highlight the importance of considering PRES as a differential diagnosis while providing care to MMD patients with concurrent acute onset of neurological symptoms.

## Author contributions

**Data curation:** Meng-Ni Wu.

**Supervision:** Ping-Song Chou.

**Visualization:** I-Hsiao Yang.

**Writing – original draft:** Chun-Hsin Teng.

**Writing – review & editing:** Ping-Song Chou.
